# The impact of the ST131 clone on recurrent ESBL-producing *E. coli* urinary tract infection: a prospective comparative study

**DOI:** 10.1038/s41598-022-14177-y

**Published:** 2022-06-16

**Authors:** Anna Lindblom, Camilla Kiszakiewicz, Erik Kristiansson, Shora Yazdanshenas, Nina Kamenska, Nahid Karami, Christina Åhrén

**Affiliations:** 1 Department of Infectious Diseases, Institute of Biomedicine, University of Gothenburg, Guldhedsgatan 10A, 413 46 Gothenburg, Sweden; 2 Department of Clinical Microbiology, Sahlgrenska University Hospital, Region Västra Götaland, Gothenburg, Sweden; 3 Centre for Antibiotic Resistance Research (CARe), University of Gothenburg, Gothenburg, Sweden; 4 Unilabs, Department of Clinical Microbiology, Skaraborg Hospital, Skövde, Sweden; 5 Department of Mathematical Sciences, Chalmers University of Technology, Gothenburg, Sweden; 6 NU Hospital Group, Department of Clinical Microbiology, Region Västra Götaland, Trollhättan, Sweden; 7 Swedish Strategic Program Against Antimicrobial Resistance (Strama), Region Västra Götaland, Gothenburg, Sweden

**Keywords:** Bladder, Infectious-disease diagnostics

## Abstract

The global emergence of extended-spectrum beta-lactamase-producing *Escherichia coli* (ESBL-*E. coli*), mainly causing urinary tract infections (UTI), is of great concern. Almost one third of patients with UTI, develop recurrent UTI (RUTI). We followed 297 patients for one year after their first episode of UTI due to ESBL-*E. coli*. Our aim was to evaluate the impact of the globally dominant sequence type (ST)131 clone and its clades, on the risk of subsequent recurrences with ESBL-*E. coli*. Isolates from patients developing RUTI (68/297) were compared with those from patients with sporadic UTI (SUTI, 229/297). No association was found between RUTI and the two most prevalent phylogroups B2 and D, *bla*_CTX-M_ genes, or resistance profile. Half of the patients with RUTI were infected with ST131 isolates. Clade C2 were in dominance (50/119) among ST131 isolates. They were more common in patients with RUTI than SUTI (28% vs 13%) and multivariate analysis showed an increased odds-ratio (OR = 2.21, p = 0.033) for recurrences in patients infected with these isolates as compared to non-ST131 isolates. Detecting specific biomarkers, as ST131 clade C2, in ESBL-*E. coli* UTI isolates may aid in prediction of RUTI and improve diagnostics and care of patients with a risk of ESBL-*E. coli* recurrences.

## Introduction

*Escherichia coli (E. coli*) is the most common cause of urinary tract infections (UTIs) and bloodstream infections^1^. The increasing antimicrobial resistance in these organisms limits therapeutic alternatives and increases morbidity and mortality^2^. Of particular concern is the production of extended-spectrum beta-lactamases (ESBLs) and carbapenemases. Recurrent UTI (RUTI) affects approximately one third of patients with UTI and thus poses an important clinical problem, considering that UTI are among the most prevalent bacterial infections worldwide^1,3,4^.

*E. coli* can be divided into seven phylogroups (A, B1, B2, C, D, E and F) and one rarely detected phylogroup, named clade I^5^. Phylogroups C, E and F are closely related to phylogroup A, D and B2, respectively. Phylogroup B2 and D in particular have been associated with extra-intestinal infections as UTI. However, isolates of all phylogroups may cause extraintestinal infections and all have the ability to colonize the intestine and be part of the gut microbiome^6^.

The emergence of the increasingly multidrug-resistant *E. coli* of sequence type (ST)131 is considered as an important cause of global spread of antimicrobial resistance in *E. coli*^7–11^. Presently, it is the most common extraintestinal pathogenic *E. coli* clone that produce ESBL worldwide^12,13^. The phenomenal success of this high-risk clone is most likely multifactorial and does not solely relay on virulence potential^13^. ST131-isolates belong to phylogroup B2 and a majority to serotype O25b:H4, often referred to as ST131-O25b^9,13^. Nowadays ST131 is divided into three major clades, A, B and C^14^. The major drivers of antimicrobial resistance are isolates from the dominating clade C which has developed into the fluoroquinolone-resistant sister clades, clade C1 and C2 (former *H*30R and *H*30Rx)^7,11,14^. Clade C2 is primarily associated with *bla*_CTX-M-15_ (part of CTX-M group 1) and C1 with *bla*_CTX-M-14_ or *bla*_CTX-M-27_ (parts of CTX-M group 9) which are the dominant ESBLs worldwide^10,15^. Clade C2 has been linked to higher rates of persistent UTI and adverse clinical outcomes, including significant association with septicemia^7,11,12^. According to a recent study from Canada, eradicating clade C2 isolates in this region would have significant impact on public health and antibiotic resistance rates in *E. coli*^12^. An additional subclade, C1-M27, part of clade C1 and associated with *bla*_CTX-M-27_, has lately emerged and is becoming globally spread^13,14,16,17^.

We recently found *H30*Rx (that is clade C2) isolates to be associated with an increased number of UTI recurrences^18^. We also reported that in the great majority (97%) of cases the same ESBL-producing *E. coli* (ESBL-*E. coli*) strain caused all subsequent UTI recurrences for up to one year, especially evident the first six months. This is in line with studies describing a bladder, a vaginal or an intestinal reservoir of *E. coli* that repeatedly can cause disease^19,20^ and is also in line with findings in studies of RUTI caused by *E. coli* not producing ESBL^6,21^. It is however unclear if the risk of recurrences can be associated with certain bacterial features or clones, especially in the case of multidrug resistant bacteria^20^.

In this study we prospectively followed patients after an episode of UTI caused by ESBL-*E. coli* for subsequent UTI recurrences caused by ESBL-*E. coli* during the following year. Isolates from patients subsequently developing RUTI were compared with those from patients with only one sporadic UTI (SUTI) episode. We aimed to extend our previous findings of RUTI due to ESBL-*E. coli* and investigate if ESBL-*E. coli* isolates part of certain clones, especially the globally dominant ST131clone and its clades could be associated with an increased risk of developing RUTI caused by ESBL-*E. coli* as compared with sporadic UTI. If so, knowledge of the clonal properties of the initial infecting ESBL-*E. coli* strain could be valuable for medical practitioners to identify at risk populations for RUTI due to ESBL-*E. coli*. This risk assessment may be extra valuable considering that the therapeutic options for ESBL-*E. coli* infections are limited and broad-spectrum antibiotics should be used cautiously not to drive antibiotic resistance development further.

## Results

### Demographic patient data

In total, 297 patients with UTI due to ESBL- *E. coli* and no previous history of ESBL- *E. coli* infection were followed prospectively. Of these, 68 (23%) patients had recurrent UTI and the remaining 229 patients had a sporadic UTI with no additional ESBL-*E. coli* UTI episodes recorded within the following 12 months (Table 1). For most patients, the first UTI episode were sampled in outpatient care, which is 57% for those with RUTI and 69% for SUTI. Women dominated irrespective of setting. Patients with subsequent RUTI were older than those with SUTI among both men (median 71 versus 67 years) and women (median 75 versus 54 years). A culture history of recurrent significant bacteriuria was not noted in patients subsequently developing RUTI prior to inclusion.

**Table 1:** Patient demographics for 297 patients in relation to sporadic or recurrent UTI caused by ESBL-producing *E. coli.*

Setting/gender	Age group (years)	Median age (range)	Recurrent UTI, n = 68 (%)	Sporadic UTI, n = 229 (%)	Total, n = 297 (%)
**Hospital care**	–	–	29 (43)	71 (31)	100 (34)
Men	All	67 (22–96)	10 (15)	24 (10)	34 (11)
	15–65	51 (22–64)	4	12	16
	> 65	76 (66–96)	6	12	18
Women	All	65 (18–95)	19 (28)	47 (21)	66 (22)
	15–65	39 (18–65)	6	28	34
	> 65	82 (67–95)	13	19	32
**Primary care**	–	–	39 (57)	158 (69)	197 (66)
Men	All	68 (16–94)	12 (17)	19 (8)	31 (10)
	15–65	54 (16–63)	4	9	13
	> 65	78 (67–94)	8	10	18
Women	All	57 (16–100)	27 (40)	139 (61)	166 (56)
	15–65	45 (16–65)	10	91	101
	> 65	78 (66–100)	17	48	65

**Figure 1:**
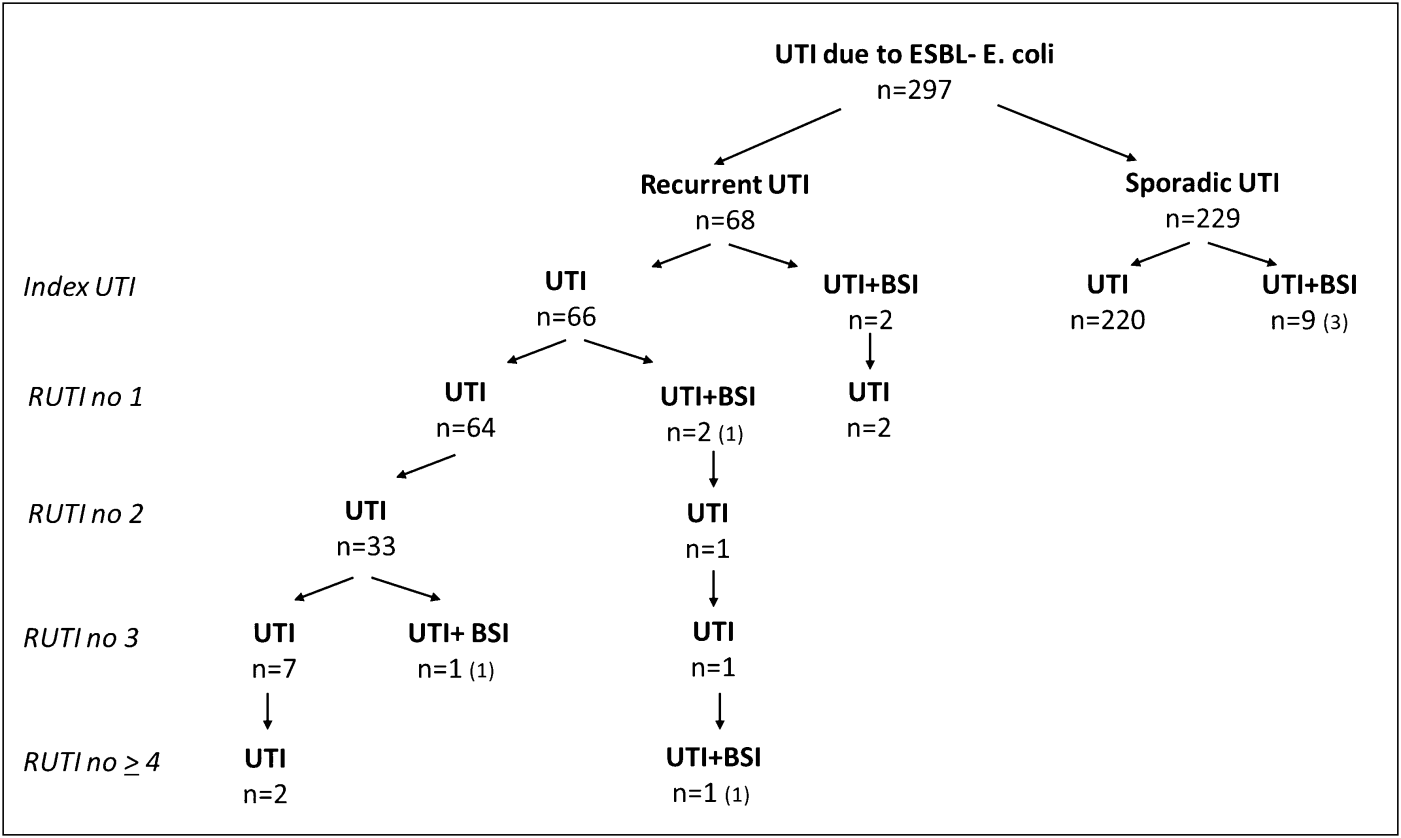
Number of patients with sporadic urinary tract infection (UTI) or recurrent UTI (RUTI) within one year after an index UTI due to ESBL-*E. coli*. All episodes are caused by ESBL- *E. coli*. Number of patients with concomitant bacteraemia (BSI) at each UTI episode are given and numbers within brackets are patients infected with ST131 clade C2.

In patients with RUTI, the median number of UTI episodes was 3 (range 2–7) and the median time to the first recurrence was 55 days (range 30–158 days). Simultaneous bacteremia was rare both in patients with RUTI and SUTI as shown in Fig. 1. The proportion of febrile and nonfebrile UTI according to referral data was estimated to be approximately 9% in patients with SUTI and 12% in patients with RUTI. Febrile UTI was only occasionally reported for the following RUTI episodes.

### Phylogroups in relation to sporadic and recurrent UTI

The isolates were typed with regard to phylogroups to obtain a general overview, grouping of isolates and to allow for historical comparison. Most isolates belonged to phylogroup B2 both from patients developing RUTI (66%) and SUTI (56%), followed by phylogroup D isolates (RUTI 15% and SUTI 21%), respectively (Table 2). Phylogroups A, B1, C, E, F and clade I were comparatively rare (3–20 isolates/phylogroup) and, except for phylogroup A, were predominantly found in isolates from patients with SUTI. The relative proportions of phylogroups were similar for isolates from patients with SUTI and RUTI and no association with RUTI was detected for the two major phylotype B2 and D (Table 3). Among phylogroup D isolates, a considerable number (49/59) were detected in patients with SUTI. A substantial part (88%) of these isolates came from women, several of them (67%, 29/43) diagnosed with SUTI in primary care.

**Table 2:** Distribution of phylogroups in 297 ESBL- *E. coli* isolates in relation to recurrent and sporadic urinary tract infections (UTI) and patient demographics.

Type of UTI/gender	Age group	Total, n = 297	Phylogroups according to the index isolates									
			A, n = 20 (%)		B1, n = 10 (%)	B2, n = 173 (%)	C, n = 4 (%)	D, n = 59 (%)	E, n = 8 (%)	F, n = 12 (%)	Clade I, n = 3 (%)	Unknown, n = 8 (%)
**Recurrent UTI**		68	9 (13)		3 (4)	45 (66)	0	10 (15)	0	1 (1)	0	0
Men	15–65	8	1		0	6 (75)	0	1	0	0	0	0
	> 65	14	1		0	12 (86)	0	0	0	1	0	0
Women	15–65	16	1		0	8 (50)	0	7 (44)	0	0	0	0
	> 65	30	6 (20)		3	19 (63)	0	2	0	0	0	0
**Sporadic UTI**		229	11 (5)		7 (3)	128 (56)	4 (2)	49 (21)	8 (3)	11 (5)	3 (1)	8 (3)
Men	15–65	21	0		0	17 (81)	0	3	1	0	0	0
	> 65	22	1		2	13 (59)	1	3	1	1	0	0
Women	15–65	119	5		0	66 (55)	2	28 (23)	4	6 (5)	3	5
	> 65	67	5		5	32 (48)	1	15 (22)	2	4	0	3

**Table 3:** Univariate regression analyses of the association of recurrent urinary tract infections with patient and bacterial characteristics in 297 patients with ESBL-*E. coli* urinary tract infection (UTI).

	Recurrent UTI, n = 68 (%)	Sporadic UTI, n = 229 (%)	Univariate regression analysis		
			Odds ratio	95% CI	p-value
**Patient characteristics**					
Increasing age			1.03	(1.01, 1.04)	0.00033
Male gender	22 (32)	43 (19)	2.07	(1.13, 3.79)	0.019
Primary care setting	39 (57)	158 (69)	0.60	(0.35, 1.05)	0.076
**Bacterial characteristics**					
Phylogroup B2	45 (66)	128 (56)	1.54	(0.88, 2.72)	0.13
Phylogroup D	10 (15)	49 (21)	0.63	(0.30, 1.33)	0.23
Sequence type (ST) 131	36 (53)	83 (36)	1.98	(1.15, 3.42)	0.014
Clade A	5 (7)	16 (7)	1.42	(0.49, 4.18)	0.52
Clade C	30 (44)	59 (26)	2.32	(1.30, 4.15)	0.0047
**Clade C1**	11 (16)	28 (12)	0.58	(0.41, 1.44)	0.15
Subclade C1-M27	7 (10)	19 (8)	1.68	(0.65, 4.33)	0.28
Subclade C1-nonM27	4 (6)	9 (4)	2.03	(0.59, 7.00)	0.26
Clade C2	19 (28)	31 (13)	2.80	(1.41, 5.56)	0.0034
Other ST131 isolates	1 (1)	8 (3)	0.57	(0.07, 4.72)	0.60

### ST131 clades in relation to sporadic and recurrent UTI

All index isolates were tested for ST131, its clades and subclades. Altogether, 53% (36/68) of the isolates in patients subsequently developing RUTI were part of ST131, and 44% (30/68) belonged to the different C clades (Table 4). In patients with SUTI, the corresponding frequencies were 36% and 26%. Clade A isolates were equally prevalent in both patient groups, that is around 7% whereas clade B isolates were rare and only detected in patients with SUTI. ST131 per se, as well as clade C, were significantly associated with RUTI in the univariate analyses, the OR being 1.98 (p = 0.014) and 2.32 (p = 0.0047) respectively (Table 3). A significant association was also seen for clade C2 but not for clade C1, subclade C1-M27 or clade A.

**Table 4:** Sequence type (ST) 131 status for the index ESBL-*E. coli* isolates in 297 patients with recurrent or sporadic urinary tract infection (UTI).

ST131 status and clades	Number of patients		
	Recurrent UTI	Sporadic UTI	Total
	n = 68 (%)	n = 229 (%)	n = 297 (%)
**ST131**	36 (53)	83 (36)	119 (40)
Clade A	5 (7)	16 (7)	21 (7)
Clade B	0	6 (3)	6 (2)
**Clade C**			
Subclade C1-M27	7 (10)	19 (8)	26 (9)
Subclade C1-nonM27	4 (6)	9 (4)	13 (4)
Clade C2	19 (28)	31 (13)	50 (17)
IL	1 (1)	2 (1)	3 (1)
Non-ST131	32 (47)	146 (64)	178 (60)

To further explore the increased risk of RUTI, we performed a multivariate analysis in which phylogenetic markers were associated with RUTI while adjusting for outpatient care, increasing age and male sex. For the respective C clades, RUTI isolates were more or equally prevalent than SUTI isolates. This was most evident for clade C2 (28% RUTI and 14% SUTI), which, apart from increasing age (OR 1.02, p = 0.0017), was the only factor significantly associated with recurrences in the multivariate analyses (OR 2.21, p = 0.033, Table 5). Patients infected with isolates part of clade C2 were found in both settings in the entire region, among both sexes and in all age-groups both for those with subsequent RUTI or SUTI. A positive blood culture simultaneously with the index UTI in patients infected with clade C2 isolates was noted in 3/31 patients with SUTI and 0/19 patients with RUTI and in 1/19 patients in association with a recurrent episode (Fig. 1). For the patients with RUTI due to clade C2, presence of clade C2 in paired isolates was confirmed in all 19 cases.

**Table 5:** Multivariate regression analyses of the association of recurrent urinary tract infections with patient characteristics and selected sequence type (ST)131 characteristics in 297 patients with ESBL-*E. coli* urinary tract infection (UTI).

	Recurrent UTI, n = 68	Sporadic UTI, n = 229	Multivariate regression analyses		
			Odds ratio	95% CI	p-value
**Patient characteristic**					
Increasing age			1.02	(1.01, 1.04)	0.0017
Male gender	22	43	1.53	(0.79, 2.96)	0.20
Primary care setting	39	158	0.76	(0.42, 1.37)	0.35
**Bacterial characteristics (reference non-ST131 isolates)**					
Clade A	5	16	1.58	(0.52, 4.78)	0.42
Clade C1	11	28	1.42	(0.62, 3.22)	0.41
Clade C2	19	31	2.21	(1.07, 4.57)	0.033
Other ST131 isolates	1	8	0.65	(0.07, 5.72)	0.70

### CTX-M groups in relation to sporadic and recurrent UTI

Overall, CTX-M group 1 (64%) and CTX-M group 9 (34%) ESBL-genes were in clear dominance. Their relative frequency was similar between isolates from men and women and irrespective of age-group and setting. With regard to CTX-M group 1 and 9 (as well as *bla*_CTX-M-27_) per se, no significant association with RUTI was found.

### Antibiotic resistance in relation to phylogroups, ST131 clades, and recurrent and sporadic UTI

In general, high rates of resistance in addition to cephalosporin resistance were observed for the isolates (Supplementary Table). Resistance to pivmecillinam and nitrofurantoin was however rare, < 2% respectively, with no differences between the two patient groups. The rates in isolates from patients subsequently developing RUTI were, in comparison with those with SUTI, 75% vs 65%, 66% vs 61%, and 50% vs 38% for ciprofloxacin, trimethoprim and tobramycin, respectively. Resistance to all three of these antibiotic groups in addition to cephalosporins (here denoted multidrug resistant) were seen in 32% and 25% of the isolate causing RUTI and SUTI, respectively. None of these differences were, however, significant. Multidrug resistance in clade C2 isolates from the two patient groups, was seen in 53% (RUTI) and 61% (SUTI). For phylogroup D isolates, the corresponding frequencies were 0% (RUTI) and 14% (SUTI) respectively. The D isolates were generally sensitive except for trimethoprim resistance (71%).

## Discussion

In this study, we prospectively followed almost 300 patients with UTI due to ESBL-*E. coli* with regard to subsequent ESBL-*E. coli* recurrences within the following year, of which 23% developed RUTI within six months. The main finding was the association of ST131, especially clade C2 with recurrent ESBL- *E. coli* UTI.

In analogy with previous studies^6,18,21^, we also demonstrate that ESBL-*E. coli* strains of most phylogroups can cause both RUTI and SUTI. As expected, isolates of phylogroup B2 and D dominated, but none of these phylogroups could be associated with an increased risk of recurrences. A substantial number of the phylogroup D isolates were detected in patients with SUTI, mostly in women and in primary care. However, our preliminary data indicates that this was a very heterogeneous group of isolates with regard to strain types.

To the best of our knowledge, there are no other studies prospectively evaluating the risk of subsequent recurrences of ESBL-*E. coli* UTI in relation to strain characteristics in a similar low-endemic setting where the risk of recolonization with new ESBL-*E. coli* is very low. The phylogroup distribution, recurrence rates and patient demographics were similar to previous studies of RUTI due to *E. coli* lacking ESBL^19,21–23^. The results for the RUTI group were also very similar to our previous study including over 100 patients with RUTI due to ESBL-*E. coli* infected several years earlier^18^, which further validates our findings. In the previous study we could demonstrate an increased number of recurrences caused by *H30*Rx isolates (that is clade C2), as compared to other types of ESBL-*E. coli* within phylogroup B2. In the present study we extend this finding by showing that clade C2 was associated with a more than two-fold increase in odds-ratio for RUTI as compared to non-ST131 ESBL-*E. coli*. This is in accordance with another Swedish study reporting an association with ST131 and subsequent recurrent infections after ESBL-*E. coli* community acquired bacteremia as compared to other STs^24^.

ESBL-*E. coli* ST131 has repeatedly been associated with elderly patients in hospitals or long-term care facilities^7,25,26^ and elderly patients were also in dominance among patients with clade C2 isolates in this study. A recent French study revealed likely community acquisition and confirmed the difficulty to identify common patient associated risk factors associated with carriage and infection with ST131 isolates^27^. These authors suggested that the superiority of ST131 may indeed rely more on bacterial factors than on host characteristics. The opportunistic properties and ability to persist in the gut of ST131 have been highlighted in association with disease development rather than its virulence properties or certain sets of virulence associated genes^12,13,23,28,29^. For instance, ST131 and the clade C2 in particular have been shown to select genes favoring anaerobic metabolism and colonization^30^, which could contribute to its ability to cause not only UTI but also RUTI, especially in a host prone to develop recurrences^30^.

It has been reported that isolates with CTX-M group 9 enzymes are carried in the gut flora for a more extended time than those with CTX-M group 1^31,32^. This could favor the ability to cause RUTI, but we found no association between CTX-M groups per se and subsequent recurrences. We found no association for RUTI and a particular antibiotic resistance or multidrug resistance profile. Interestingly, multidrug resistance was more common, however not significantly, in isolates from patients with SUTI than RUTI, both for ST131 clade C2 and for the phylogroup D isolates.

The present study has limitations. Since only referral data and not entire medical records have been studied, misclassification, for instance of asymptomatic bacteriuria as UTI, cannot be completely excluded. Nor could we exclude that patient factors predisposing for RUTI could have selected for infection with clade C2 isolates. These isolates were, however detected in both sexes, all age-groups and in patients seeking care both in- and outpatient settings distributed over a high number of caregivers in a large region. This indicates that the clone is generally spread among different patient categories and in our entire region. Also, the urine culture history the year prior to inclusion did not confirm a previous history of RUTI which could have indicated preexisting patient factors favoring RUTI in patients infected with this subclone. Patients with febrile UTI were in minority in the entire study suggesting that presence of bacteremia or pyelonephritis did not influence the occurrence of RUTI in the present study.

The general recommendation for urine cultures in Sweden does not include first-time UTI in otherwise healthy women meaning that urine cultures could be missing and that isolates in this study might not be totally representative for patients with SUTI. Nevertheless, all ESBL-*E. coli* urinary isolates were saved during the entire study period to assure that a minimum of isolates was lost. Confirmation of strain identity in the following episode in patients with RUTI was not made as several studies, including our previous study^18,20,23^, have shown high concordance in paired isolates from patients with recurrent *E. coli* infections including ESBL-*E. coli*. To once again ascertain this prerequisite for our findings paired isolates from all patients with RUTI due to clade C2 isolates were compared demonstrating identity.

It is generally believed that the patients’ own fecal and vaginal flora acts as a reservoir for infecting *E coli* strains that cause UTI including RUTI^19,20,29^. It has also been shown that *E. coli* that cause recurrences can persist in the bladder epithelium and form biofilm-like intracellular communities, thereby serving as a reservoir for RUTI^33,34^. Antibiotic treatment has been associated with avoidance of intestinal clearance of the bacteria including ST131 and clade C2 isolates^28^. We cannot exclude the possibility that antibiotic treatment has contributed to the persistence of this subclade in the gut flora favoring the ability to cause subsequent recurrent UTI episodes. Nevertheless, compliance to first line treatment choice for lower UTI irrespective of sex (including ESBL-*E. coli*) with pivmecillinam or nitrofurantoin, is very high in Sweden (https://www.folkhalsomyndigheten.se). A recent very large Danish study of *E. coli* UTI, including ESBL-*E. coli* could not associate a particular treatment choice, including these two drugs, with clinical treatment failure^35^.

Conclusions have been drawn merely from the grouping of isolates by phylogroup analysis, ST131 clades and CTX-M groups and resistance rates. We cannot exclude that other clones or clades, for instance the C1-M27 subclade, could be of importance if it continuous to emerge^17,36^. The B2-isolates not part of ST131, on the other hand, are known to be heterogeneous with no strain type in dominance^9,37^. To determine the actual CTX-M-genes or STs is unlikely to add additional value in evaluating the risk of RUTI in the present study, considering that the majority of isolates associated with RUTI were part of phylogroup B2 and ST131. Furthermore, a recent large study using whole genome sequencing could not find any association between RUTI and certain genotypes or virulence traits^23^, why additional factors involved in the pathogenesis of RUTI due to ESBL-*E. coli* remains to be explored.

## Conclusion

Our results demonstrate the association of the *E. coli* ST131 clade C2 with ESBL- *E. coli* RUTI with a two-fold increased odds ratio in patients infected with this globally prevalent clade as compared to other ESBL-*E. coli* not part of ST131. It has been found that clade C2 is responsible for the majority of cases of mismatched empirical antibiotic therapy and a rapid test to detect C2 isolates in urine samples has been proposed^38^. Monitoring the C2 clade in ESBL- *E. coli* causing UTI could be of aid when assessing the future risk of ESBL-*E. coli* RUTI in infected patients as well as to avoid transmission of these isolates between those at risk of developing disease. Considering its prevalence, we suggest that clade C2 is monitored in the routine clinical setting. The present multiplex PCR can be exchanged with an easier method, preferably a single PCR developed for this purpose. With the emergence of new ESBL- *E. coli* clones, locally or globally, their association with RUTI must also be explored.

## Patients and methods

### Patients and isolates

Between 1/10 2017 and 1/10 2018 all urinary ESBL- *E. coli* isolates from patients ≥ 15 years were collected from all clinical microbiology laboratories in Region Västra Götaland in Western Sweden (approximately 1.7 million inhabitants). There are four laboratories in the region: Sahlgrenska University Hospital (SU), Skaraborg Hospital, Södra Älvsborg Hospital and the NU-hospital group. They analyze approximately 60,000, 70,000, 20,000, and 30,000 urine samples/year, respectively, detecting *E. coli* in ~ 20% of the samples. ESBL-*E. coli* ranged between 3.8 and 6.3% among *E. coli.*

The laboratory databases were searched for urine samples positive for ESBL-*E. coli* from the in- and out-patient settings. Epidemiological and clinical data linked to the index ESBL-*E. coli* isolates were extracted from referral data as well for all subsequent UTI episodes with ESBL-*E. coli* for 1 year following the index episode. Febrile or nonfebrile UTI was evaluated from referral data and culture results for each UTI episode by experienced physicians (AL and CK). Blood cultures positive for ESBL-*E. coli* were also noted as well as a history of significant bacteriuria the preceding year. Only patients with no previous recorded history of ESBL-producing bacteria in any type of clinical or screen culture were included in the study. Only voided samples with a significant number of ESBL-*E. coli* (≥ 10^5^ CFU/ml) in monoculture were included. Cultures with isolates of more than one bacterial species were excluded, as were cultures with referral data indicating control purposes or presence of a urinary catheter or urinary abnormalities. Patients who died or moved from the region during the year following their first infection (n = 20) were excluded.

RUTI was defined according to the clinical international definition (www.uroweb.org/guideline/urological-infections/) that is two UTI within 6 months or three within a year (if the first recurrence occurs later than 6 months). Only three patients in the entire study cohort meet the latter definition. We decided not to include these patients as the clinical definition does not take strain identity in recurrent episodes into account. Urine samples with ESBL- *E. coli* positive cultures within 30 days from a previous episode were considered the same infectious episode and not included. If a urine culture with other uropathogens than ESBL- *E. coli*, including *E. coli* not producing ESBL, was detected between ESBL- *E. coli* RUTI episodes this patient was not included*.* SUTI was defined as a single urinary culture with ESBL-producing *E. coli* with no additional ESBL-*E. coli* cultures within one year. In total, 4–5 sporadic cases with ESBL-*E. coli* per week evenly distributed during the study period and laboratories were included in the sporadic group.

### Laboratory procedure

*E. coli* isolates were identified according to routine clinical microbiology practice. Antibiotic sensitivity testing was performed according to routine clinical procedure in Sweden that is using the disc diffusion method and breakpoints according to European Committee on Antimicrobial Susceptibility Testing (EUCAST) (http://www.eucast.org) at the time. Cephalosporin-resistant isolates were screened for the ESBL-phenotype using the double-disc diffusion assay^39^ and later genetically confirmed. ESBL-positive isolates were stored at −70 °C, and subsequently analyzed at SU. Frozen isolates were retrieved and incubated overnight at 37 °C on blood agar. From the bacterial culture, DNA was extracted as previously described^40^. The data presented are based on the results analyzing the index isolates with the exception of the 19 patients with RUTI due to ST131 clade C2 where paired isolates were analyzed for homology with PCR for this clade as outlined below.

### Determination of ST131 clades and phylogroups

The ST131 clades; A, B, C, C2 and the two clusters of clade C1 (subclade C1-M27 and the C1-nonM27 isolates) were established using the PCR assay developed by Matsumura et al.^16^. *E. coli* phylogroups were determined using the updated Clermont method^5^.

### Detection of ESBL genes

All isolates were investigated for the CTX-M groups 1, 2, and 9, using a Taq-Man PCR protocol^41^. Negative isolates were subsequently tested for the TEM, OXA and SHV genes, as described^40^.

### Statistical analysis

The association between the risk of RUTI and covariates, including phylogroup B2 and D (yes/no), ST131 (yes/no), its respective clades and subclades (yes/no), outpatient care (yes/no), age and sex, was assessed using logistic regression model with the canonical logit link. Age was analyzed as a continuous variable presented as increase in odds ratio per patient year. The statistical analysis was done both in a univariate setting, where each covariate was assessed individually, and in a multivariate setting taking outpatient care, increasing age and male sex into account. In both settings, the difference in odds ratio (OR) for RUTI between ST131-positive and ST131-negative isolates were compared. For all analyses, confidence intervals were calculated at 95% using normal approximations. Significance for individual covariates were assessed using Wald tests. Tests with p-values less than 0.05 were considered significant.

### Ethical approval

This study was approved by the Regional Ethical Review Board in Gothenburg, Sweden (Permit no. 170-17).

### Informed consent statement

Informed consent was not applicable as the study primarily involved bacteria in accordance with the approval of the ethical review board.

## Supplementary Information

Supplementary Tables.

## Data Availability

The datasets used and/or analyzed during the current study are available from the corresponding author on reasonable request.

## References

[1] Foxman B (2014). Urinary tract infection syndromes: Occurrence, recurrence, bacteriology, risk factors, and disease burden. Infect. Dis. Clin. N. Am..

[2] Shamsrizi P (2020). Variation of effect estimates in the analysis of mortality and length of hospital stay in patients with infections caused by bacteria-producing extended-spectrum beta-lactamases: A systematic review and meta-analysis. BMJ Open.

[3] Hooton TM (2012). Clinical practice. Uncomplicated urinary tract infection. N. Engl. J. Med..

[4] Lindblom A, Karami N, Magnusson T, Ahren C (2018). Subsequent infection with extended-spectrum beta-lactamase-producing Enterobacteriaceae in patients with prior infection or fecal colonization. Eur. J. Clin. Microbiol. Infect. Dis..

[5] Clermont O, Christenson JK, Denamur E, Gordon DM (2013). The Clermont *Escherichia coli* phylo-typing method revisited: Improvement of specificity and detection of new phylo-groups. Environ. Microbiol. Rep..

[6] Nielsen KL, Dynesen P, Larsen P, Frimodt-Moller N (2014). Faecal Escherichia coli from patients with *E. coli* urinary tract infection and healthy controls who have never had a urinary tract infection. J. Med. Microbiol..

[7] Johnson JR (2016). *Escherichia coli* sequence type 131 H30 is the main driver of emerging extended-spectrum-beta-lactamase-producing *E. coli* at a tertiary care center. mSphere..

[8] Mathers AJ, Peirano G, Pitout JD (2015). The role of epidemic resistance plasmids and international high-risk clones in the spread of multidrug-resistant Enterobacteriaceae. Clin. Microbiol. Rev..

[9] Nicolas-Chanoine MH, Bertrand X, Madec JY (2014). *Escherichia coli* ST131, an intriguing clonal group. Clin. Microbiol. Rev..

[10] Petty NK (2014). Global dissemination of a multidrug resistant *Escherichia coli* clone. Proc. Natl. Acad. Sci. U S A.

[11] Price LB (2013). The epidemic of extended-spectrum-beta-lactamase-producing *Escherichia coli* ST131 is driven by a single highly pathogenic subclone, H30-Rx. MBio.

[12] Peirano G (2020). Trends in population dynamics of *Escherichia coli* sequence type 131, Calgary, Alberta, Canada, 2006–2016(1). Emerg. Infect. Dis..

[13] Pitout JDD, Finn TJ (2020). The evolutionary puzzle of *Escherichia coli* ST131. Infect. Genet. Evolut. J. Mol. Epidemiol. Evolut. Genet. Infect. Dis..

[14] Pitout JD, DeVinney R (2017). *Escherichia coli* ST131: A multidrug-resistant clone primed for global domination. F1000Res.

[15] Bevan ER, Jones AM, Hawkey PM (2017). Global epidemiology of CTX-M beta-lactamases: Temporal and geographical shifts in genotype. J. Antimicrob. Chemother..

[16] Matsumura Y (2017). Rapid identification of different *Escherichia coli* sequence type 131 clades. Antimicrob. Agents Chemother..

[17] Merino I (2018). Emergence of ESBL-producing *Escherichia coli* ST131-C1-M27 clade colonizing patients in Europe. J. Antimicrob. Chemother..

[18] Karami N, Lindblom A, Yazdanshenas S, Linden V, Ahren C (2020). Recurrence of urinary tract infections with extended-spectrum beta-lactamase-producing *Escherichia coli* caused by homologous strains among which clone ST131-O25b is dominant. J. Glob. Antimicrob. Resist..

[19] Moreno E (2008). Relationship between *Escherichia coli* strains causing acute cystitis in women and the fecal *E. coli* population of the host. J. Clin. Microbiol..

[20] Thanert R (2019). Comparative genomics of antibiotic-resistant uropathogens implicates three routes for recurrence of urinary tract infections. MBio.

[21] Ejrnaes K (2011). Characteristics of *Escherichia coli* causing persistence or relapse of urinary tract infections: Phylogenetic groups, virulence factors and biofilm formation. Virulence.

[22] Johnson JR (2005). Extended virulence genotypes and phylogenetic background of *Escherichia coli* isolates from patients with cystitis, pyelonephritis, or prostatitis. J. Infect. Dis..

[23] Nielsen KL (2021). *Escherichia coli* causing recurrent urinary tract infections: Comparison to non-recurrent isolates and genomic adaptation in recurrent infections. Microorganisms..

[24] Froding I (2020). Extended-spectrum-beta-lactamase- and plasmid AmpC-producing *Escherichia coli* causing community-onset bloodstream infection: Association of bacterial clones and virulence genes with septic shock, source of infection, and recurrence. Antimicrob. Agents Chemother..

[25] Holland MS (2020). Molecular epidemiology of *Escherichia coli* causing bloodstream infections in a centralized Canadian region: A population-based surveillance study. Clin. Microbiol. Infect..

[26] Overdevest I (2016). Prolonged colonisation with *Escherichia coli* O25:ST131 versus other extended-spectrum beta-lactamase-producing *E. coli* in a long-term care facility with high endemic level of rectal colonisation, the Netherlands, 2013 to 2014. Euro Surveill..

[27] Muller A (2021). Hospital-diagnosed infections with *Escherichia coli* clonal group ST131 are mostly acquired in the community. Sci. Rep..

[28] Johnson JR (2016). The pandemic H30 subclone of *Escherichia coli* sequence type 131 is associated with persistent infections and adverse outcomes independent from its multidrug resistance and associations with compromised hosts. Clin. Infect. Dis..

[29] Nielsen KL (2017). Whole-genome comparison of urinary pathogenic *Escherichia coli* and faecal isolates of UTI patients and healthy controls. Int. J. Med. Microbiol..

[30] Alqasim A, Scheutz F, Zong Z, McNally A (2014). Comparative genome analysis identifies few traits unique to the *Escherichia coli* ST131 H30Rx clade and extensive mosaicism at the capsule locus. BMC Genomics.

[31] Arcilla MS (2017). Import and spread of extended-spectrum beta-lactamase-producing Enterobacteriaceae by international travellers (COMBAT study): A prospective, multicentre cohort study. Lancet Infect. Dis..

[32] Titelman E (2014). Faecal carriage of extended-spectrum beta-lactamase-producing Enterobacteriaceae is common 12 months after infection and is related to strain factors. Clin. Microbiol. Infect..

[33] Kerrn MB, Struve C, Blom J, Frimodt-Moller N, Krogfelt KA (2005). Intracellular persistence of *Escherichia coli* in urinary bladders from mecillinam-treated mice. J. Antimicrob. Chemother..

[34] Mulvey MA, Schilling JD, Hultgren SJ (2001). Establishment of a persistent *Escherichia coli* reservoir during the acute phase of a bladder infection. Infect. Immun..

[35] Jansaker F (2019). Pivmecillinam compared to other antimicrobials for community-acquired urinary tract infections with *Escherichia coli*, ESBL-producing or not—A retrospective cohort study. Infect Drug Resist.

[36] Matsumura Y (2016). Global *Escherichia coli* sequence type 131 clade with blaCTX-M-27 gene. Emerg. Infect. Dis..

[37] Brisse S (2012). Phylogenetic distribution of CTX-M- and non-extended-spectrum-beta-lactamase-producing *Escherichia coli* isolates: Group B2 isolates, except clone ST131, rarely produce CTX-M enzymes. J. Clin. Microbiol..

[38] Tchesnokova V, Riddell K, Scholes D, Johnson JR, Sokurenko EV (2019). The uropathogenic *Escherichia coli* subclone sequence type 131–H30 is responsible for most antibiotic prescription errors at an urgent care clinic. Clin. Infect. Dis..

[39] Drieux L, Brossier F, Sougakoff W, Jarlier V (2008). Phenotypic detection of extended-spectrum beta-lactamase production in Enterobacteriaceae: Review and bench guide. Clin. Microbiol. Infect..

[40] Karami N, Helldal L, Welinder-Olsson C, Ahren C, Moore ER (2013). Sub-typing of extended-spectrum-beta-lactamase-producing isolates from a nosocomial outbreak: Application of a 10-loci generic *Escherichia coli* multi-locus variable number tandem repeat analysis. PLoS ONE.

[41] Birkett CI (2007). Real-time TaqMan PCR for rapid detection and typing of genes encoding CTX-M extended-spectrum beta-lactamases. J. Med. Microbiol..

