# Supplementary material for: The impact of the ST131 clone on recurrent ESBL-producing E. coli urinary tract infection: a prospective comparative study
Source: Sci Rep. 2022 Jun 16;12:10048. doi: 10.1038/s41598-022-14177-y (PMC9203711; doi:10.1038/s41598-022-14177-y)
Supplement: Supplementary file 1 — Supplementary Tables. [file 41598_2022_14177_MOESM1_ESM.docx]

|  | **Recurrent ESBL-*E. coli*** | Resistance to | | | | **Sporadic ESBL-*E. coli*** | Resistance to | | | | |  |
| --- | --- | --- | --- | --- | --- | --- | --- | --- | --- | --- | --- | --- |
| Phylogroup/ST131 status | n=68 | CIP  n=51 | TMP  n=45 | TOB  n=34 | MultiR  n=22 | n=229 | CIP  n=149 | TMP  n=139 | TOB  n=87 | | MultiR  n=58 |  |
| A | 9 | 7 | 6 | 5 | 4 | 11 | 6 | 6 | 5 | | 4 |  |
| B1 | 3 | 0 | 3 | 0 | 0 | 7 | 4 | 4 | 2 | | 1 |  |
| B2  ST131  Clade A  Clade B  Subclade C1-M27  Subclade C1-nonM27  Clade C2  I1  Not ST131 | 45  36  5  0  7  4  19  1  9 | 41  36  5  0  7  4  19  1  5 | 28  24  4  0  5  3  11  1  4 | 28  24  3  0  0  4  17  0  4 | 17  15  2  0  0  3  10  0  2 | 128  83  16  6  19  9  31  2  45 | 99  72  11  2  19  9  31  0  27 | 74  49  12  2  13  3  19  0  25 | 57  46  10  1  0  6  29  0  11 | 36  30  8  1  0  2  19  0  6 | | |
| C | 0 | 0 | 0 | 0 | 0 | 4 | 2 | 3 | 2 | | 2 |  |
| D | 10 | 2 | 7 | 0 | 0 | 49 | 15 | 35 | 9 | | 7 |  |
| E | 0 | 0 | 0 | 0 | 0 | 8 | 7 | 5 | 5 | | 3 |  |
| F | 1 | 1 | 1 | 1 | 1 | 11 | 10 | 6 | 5 | | 4 |  |
| Clade I | 0 | 0 | 0 | 0 | 0 | 3 | 3 | 2 | 1 | | 1 |  |
| Unknown | 0 | 0 | 0 | 0 | 0 | 8 | 3 | 4 | 1 | | 0 |  |

**Supplementary table.** Antibiotic resistance against ciprofloxacin (CIP), trimethoprim (TMP) and tobramycin (TOB) respectively and to more than three antibiotic groups (MultiR) in relation to phylogroup, sequence type (ST)131 status, in ESBL-*E. coli* from 297 patients with recurrent or sporadic urinary tract infection (UTI)
